# Application of lactic acid bacteria producing antifungal substance and carboxylesterase on whole crop rice silage with different dry matter

**DOI:** 10.5713/ajas.20.0545

**Published:** 2020-10-14

**Authors:** Seong Shin Lee, Dimas Hand Vidya Paradhipta, Hyuk Jun Lee, Young Ho Joo, Hyeon Tak Noh, Jeong Seok Choi, Keum Bae Ji, Sam Churl Kim

**Affiliations:** 1 Division of Applied Life Science (BK21Four, Insti. of Agri. & Life Sci.), Gyeongsang National University, Jinju 52828, Korea; 2 Faculty of Animal Science, Universitas Gadjah Mada, Yogyakarta 55281, Indonesia; 3 Institute of Technology, Livemac Co. Ltd., Gimje 54329, Korea

**Keywords:** Haylage, Lactic Acid Bacteria, Rumen Fermentation, Silage, Whole Crop Rice

## Abstract

**Objective:**

This study was conducted to investigate effects of antifungal substance and carboxylesterase-producing inoculant on fermentation indices and rumen degradation kinetics of whole crop rice (WCR) silage ensiled at different dry matter (DM) contents.

**Methods:**

Dual-purpose inoculants, *Lactobacillus brevis* 5M2 and *Lactobacillus buchneri* 6M1, confirmed both activities of antifungal and carboxylesterase in the previous study. The WCR at mature stage was chopped, and then wilted to obtain three different DM contents consisting of 35.4%, 43.6%, and 51.5%. All WCR forages were applied distilled water (CON) or mixed inoculants with 1:1 ratio at 1×10^5^ colony forming unit/g (INO), and ensiled into 20 L mini silo (5 kg) in quadruplicates for 108 d.

**Results:**

The INO silages had lower lactate (p<0.001) and butyrate (p = 0.022) with higher acetate (p<0.001) and propionate (p<0.001) than those of CON silages. Ammonia-N (p<0.001), lactate (tendency; p = 0.068), acetate (p = 0.030), and butyrate (p<0.001) concentrations of INO silages decreased linearly with increasing DM content of WCR forage. The INO silages presented higher lactic acid bacteria (p<0.001) with lower molds (p< 0.001) than those of CON silages. Yeasts (p = 0.042) and molds (p = 0.046) of WCR silages decreased linearly with increasing DM content of WCR forage. In the rumen, INO silages had higher the total degradable fraction (p<0.001), total volatile fatty acid (tendency; p = 0.097), and acetate (p = 0.007), but lower the fractional degradation rate (p = 0.011) and propionate (p<0.001) than those of CON silage. The total degradable fraction (p<0.001), total volatile fatty acid (p = 0.001), iso-butyrate (p = 0.036), and valerate (p = 0.008) decreased linearly with increasing DM content of WCR forage, while the lag phase (p<0.001) was increased linearly.

**Conclusion:**

This study concluded that application of dual-purpose inoculants on WCR silage confirmed antifungal and carboxylesterase activities by inhibiting mold and improving rumen digestibility, while increase of wilting times decreased organic acids production and rumen digestibility.

## INTRODUCTION

An inhibition of undesirable microbes and an increase of nutrient digestibility are the main foci in silage studies. The growths of yeast and mold decrease the nutrient quality of silage and produce harmful substances for ruminants [1]. In addition, digestibility of silage decreases with increasing lignocellulose content. Rumen microbes are capable of producing cellulase or hemicellulase, but they release limited enzymes to breakdown lignocellulose [2]. The previous investigations revealed that several strains of lactic acid bacteria (LAB) could produce antifungal substances to inhibit growth undesirable microbes [3–5] and carboxylesterase to break down lignincellulose [1,4,5]. However, the ability of LAB to produce antifungal or carboxylesterase activities during ensiling were depended on strains and substrate sources [1,6].

In South-East Asia, rice straw is abundant and has become one of the biggest forage sources for feeding ruminants, such as cattle, goat, sheep, and even buffaloes [7]. After harvesting, the rice straw commonly is preserved by making silage or hay. However, it has poor content of soluble carbohydrate after separating from its grains, which results in a low fermentation quality when it is preserved as silage [8]. The LAB cannot grow optimally with low content of soluble carbohydrate leading an inability to drop the pH rapidly during ensiling [9]. In addition, the maillard reaction and an increase of yeast and mold can occur as the consequence of low fermentation quality in the silage [9]. Ensiling rice straw together with its grains provides more water soluble carbohydrate that can increase organic acid concentration produced by LAB, and thus improve fermentation quality. Even though the fermentation quality can be increased, whole crop rice (WCR) at maturity stage contains a lot of lignocellulose that decreases digestibility. Thus, appropriate LAB producing antifungal and esterase activities are required to improve fermentation and digestibility of WCR silage.

In our previous study, new dual-purpose inoculants consisting of *Lactobacillus brevis* (*L. brevis*) 5M2 KACC 92268P and *Lactobacillus buchneri* (*L. buchneri*) 6M1 KACC 92269P were obtained from corn silages ensiled at beef cattle farms through a bacterial strain isolation procedure based on antifungal activity against *Fusarium graminearum* and carboxylesterase activity [4]. In the application, isolation of baled-corn silage with mixed those LAB confirmed an antifungal effect by decreasing yeast contamination and a carboxylesterase effect by increasing nutrient digestibility in the rumen [4]. However, the effects of the isolated LAB in high lignified forage such as WCR were not tested. In hypothesis, application of new dual-purpose inoculants on WCR silage can decrease the contamination of undesirable microbes and increase nutrient digestibility in the rumen, but the effectiveness of antifungal and esterase activities may also be affected by dry matter (DM) content at ensiling. To investigate the DM effect, WCR forage was wilted in different times that represented the moisture content for making silage, haylage, and the intermediate condition between silage and haylage. Therefore, the purpose of the present study was to investigate the effect of new dual-purpose inoculant on fermentation indices and rumen degradation kinetics of WCR silage with different wilting times.

## MATERIALS AND METHODS

### Inoculant preparation

The *L. brevis* 5M2 KACC 92268P and *L. buchneri* 6M1 KACC 92269P (Korean Culture Center of Microorganism, Seoul, Korea) were grown respectively in lactobacilli MRS media at 30°C for 3 d in a CO_2_ incubator, and then adjusted with sterile ultra-pure distilled water to obtain 1×10^5^ colony forming unit (cfu)/g of fresh forage as the recommended application rate [4,5]. Furthermore, these LABs were mixed at ratio 1:1 for silage inoculant.

### Silage production

The WCR (Sindongjin hybrid) at mature stage was harvested from 2 ha paddy field at Animal Research Unit, Gyeongsang National Univeristy (Geumgok, Jinju, Gyeongnam, Korea) and chopped into 3 to 5 cm length using a conventional harvester (BHC-90; BUHEUNG Machinery Ltd., Jinju, Korea). The chopped WCR was wilted under the sun in different times to get specific DM contents following: i) low DM content, without wilting (LOW) containing 35.4% DM; ii) intermediate DM content, wilted to obtain 43.7% DM (MED); and iii) high DM content, wilted to obtain 51.6% DM (HIGH). The LOW and HIGH represented condition for making silage and haylage, respectively. The MED represented intermediate condition between silage and haylage. All forages were ensiled into 20 L mini silo (5 kg) for 108 d and were applied 1% distilled water in fresh forage (CON) or mixture of inoculants (INO). Each experimental treatment had four silos as replication, thus a total of 24 silos was used in the present study. The WCR before and after ensiling were sub-sampled at 500 g, respectively, to analyses chemical composition and *in vitro* digestibility. Also, 20 g of ensiled WCR was sub-sampled and blended with 200 mL of sterile ultrapure water for 30 s, and then filtered through two layers of cheesecloth to make silage extraction [3–5]. The fresh silage extraction was used to analyze pH and microbial counts. After then, silage extraction was stored at −70°C until analyses of ammonia-N, lactate, and volatile fatty acid (VFA).

### Chemical compositions

Forage and silage samples were dried at 65°C for 48 h and ground to pass 1-mm screen using a cutting mill (Shinmyung Electric Co., Ltd, Gimpo, Korea) for the measurements of chemical compositions and *in vitro* digestibility at 48 h, respectively. DM was determined by drying 10 g of sample in an forced-draft oven (OF-22GW; Jeio Tech, Seoul, Korea) at 105°C for 24 h and crude ash (CA) was determined with a muffle furnace at 550°C for 5 h. Crude protein (CP) and ether extract (EE) were determined by Kjeldahl (method 984.13 [10]) using N analyzer (B-324, 412, 435, and 719 S Titrino; BUCHI, Flawil, Switzerland) and Soxhlet (method 920.39; [10]), respectively. Following the procedure of AOAC [10], neutral detergent fiber (NDF; method 2002.04) and acid detergent fiber (ADF; method 973.18) were determined by using an Ankom^200^ Fiber Analyzer (Ankom Technology, Macedon, NY, USA). Hemicellulose (HEMI) was determined by calculating the differences between NDF and ADF.

### Fermentation indices

Silage pH and ammonia-N were measured using pH meter (SevenEasy; Mettler Toledo, Greifensee, Switzerland) and the colorimetric method described by Chaney and Marbach [11], respectively. The silage extraction was centrifuged at 5,645×*g* for 15 min to separate supernatant from silage residue. Collected supernatant was used for lactate and VFA analyses. The concentrations of lactate and VFA were determined using HPLC (L-2200; Hitachi, Tokyo, Japan) fitted with a UV detector (L-2400; Hitachi, Tokyo, Japan) and a column (Metacarb 87H; Varian, Palo Alto, CA, USA) according to the method described by Muck and Dickerson [12].

### Microbial counts

Silage extract (first dilution) was continued in several dilutions (10^−3^ to 10^−8^) to determine microbial counts such as LAB, yeast, and mold. The silage extract was plated in triplicate selective agar medium. The lactobacilli MRS agar media (MRS; Difco, Detroit, MI, USA) was used for LAB count, and potato dextrose agar (PDA; Difco, USA) was used for yeast and mold counts. The MRS agar plates were placed in a CO_2_ incubator (Thermo Scientific, Waltham, MA, USA) at 28°C for 48 h, while PDA plates were incubated at 28°C for 72 h in an aerobic incubator (Johnsam Corp., Boocheon, Korea) [3–5]. Visible colonies were counted from the plates and the number of cfu was expressed per gram of silage. The microbial data was transformed to log10.

### Rumen incubation

The procedure of animal care was approved by animal ethical committee of Gyeongsang National University, Jinju, Korea (GNU-191011-E0050). The rumen fluid was collected from two non-pregnant cannulated Hanwoo heifers before morning feeding, their diets consisted of rice straw and commercial concentrate mix at 8:2 ratio. The collected rumen fluid was composited, and then filtered via two layers of cheesecloth. A rumen buffer was made by mixing rumen fluid with anaerobic culture medium at 1:2 ratio described by Adesogan et al [13]. Dried sample of WCR silage at 0.5 g was weigh into incubation bottle with 40 mL of rumen buffer. Then the incubation bottle was gassed with CO_2_ and closed tightly to reach anaerobic condition. Three replications for each treatment were used along with two blanks. Gas pressure was monitored in a computer every 30 min during 72 h using wireless automated system by ANKOM^RF^ (ANKOM Technology, USA) to calculate degradation kinetics in the rumen [13]. These kinetics were generated from the gas pressure using nonlinear regression procedure of Statistical Analysis Sofware [14] to fit with the model of McDonald [15] following:

$$\text{Y}=\text{A}+\text{B }(1-{\text{e}}^{-\text{c}(\text{t}-\text{L})})\;\text{for t}>\text{L}$$

where A is the immediately degradable fraction; B is the potentially degradable fraction; A+B is total degradable fraction; C is the fractional degradation rate; L is the lag phase; and t is time of incubation (h).

After incubation, bottles were opened and transferred to 50 mL conical tubes to separate remains sample and supernatant (rumen buffer) through centrifugation at 2,568×*g* for 15 min (Supra 21k; Hanil Electric Corporation, Seoul, Korea, with rotor A50S-6C No.6). The supernatant was used to analyze rumen fermentation indices such as pH, ammonia-N, and VFA [4,5]. The procedure for analyses of pH, ammonia-N, and VFA were same as described in previous section of materials and methods.

### Statistical analysis

The experiment was conducted by a 2 (Inoculant; CON vs INO)×3 (DM content; LOW vs MED vs HIGH) factorial design with four replicates per treatment and all data were analyzed using PROC MIXED of statistical analysis system (SAS), version 9.3 package program [14] to test the effects of inoculant, DM content, and its interaction (inoculant×DM content). The model was *Y_ijk_* = *μ*+*α_i_*+*β_j_*+(*αβ*)*_ij_*+*e_ijk_*, where *Y_ijk_* = response variable, *μ* = overall mean, *α_i_* = the effect of inoculant treatment, *β_j_* = the effect of DM content treatment, (*αβ*)*_ij_* = the interaction effect of inoculant and DM content, *e_ijk_* = error term. In addition, orthogonal coefficients for linear and quadratic contrast were adjusted to account for the unequal spacing of final DM concentration (35.4% vs 43.6% vs 51.5%) with PROC IML before testing polynomial contrast with PROC GLM by SAS for significance [14]. The significant differences were declared at p≤0.05.

## RESULTS

### Chemical compositions of forage and silage

There were no differences between CON forage and INO forage on all chemical compositions before ensiling (Table 1). Also, the increasing DM content of WCR forage had no effect on CP, CA, NDF, ADF, and HEMI before ensiling, except EE content that decreased linearly (p<0.001). The mean contents of CP, EE, CA, NDF, ADF, and HEMI from fresh WCR in the present study were 8.82%, 2.53%, 7.70%, 50.1%, 28.0%, and 22.1%, respectively. The application of inoculant had no effects on the chemical compositions of WCR silage (Table 2). However, EE contents of WCR silage decreased linearly (p<0.001) with increasing DM content.

### Fermentation indices of silage

The INO silages had higher pH (p = 0.025; 4.77 vs 4.65) and concentrations of acetate (p<0.001; 7.19% vs 3.20%) and propionate (p<0.001; 0.48% vs 0.00%) than those of CON silages, but had lower concentrations of lactate (p<0.001; 3.42% vs 5.13%) and butyrate (p = 0.022; 0.51% vs 1.24%) and the ratio of lactate to acetate (p<0.001; 0.48 vs 1.76) (Table 3). The silage pH increased linearly (p = 0.021) with increasing DM content of WCR forage. On the other side, concentrations of ammonia-N (p<0.001), lactate (tendency; p = 0.068), acetate (p = 0.030), and butyrate (p<0.001) decreased linearly with increasing DM content of WCR forage. An interaction effect between inoculant and DM content was observed in the butyrate (p = 0.024), where DM content showed an effect in CON silages.

### Microbial counts of silage

In microbial counts, INO silages presented higher LAB count (p<0.001; 7.98 vs 7.23 log10 cfu/g) with lower mold count (p<0.001; not detected vs 3.15 log10 cfu/g) than CON silage (Table 4). Yeast count of WCR silage was not affected by inoculant application in the present study. However, yeast count decreased linearly (p = 0.042) with increasing DM content of WCR forage. On the other side, linear effect (p = 0.046) with increasing DM was reported to decrease mold count in the present study.

### Rumen degradation kinetics

*In vitro* rumen incubation for 72 h showed that INO silages had a higher potentially degradable fraction (p = 0.001; 3.87 vs 3.61 mL/g) and a total degradable fraction (p<0.001; 4.53 vs 4.40 mL/g), but a lower immediately degradable fraction (p = 0.032; 0.67 vs 0.79 mL/g) and the fractional degradation rate was 0.07 vs 0.08 (p = 0.011) compared with the CON silages (Table 5). On the other side, the potentially degradable fraction (p = 0.024) and the total degradable fraction (p = 0.011) of WCR silage decreased linearly with increasing DM content of WCR forage, while the lag phase increased linearly (p<0.001).

### Rumen fermentation characteristics

In rumen fermentation characteristics, INO silages had higher concentrations of total VFA (tendency; p = 0.097; 231.7 vs 229.4 m*M*/L) and acetate (p = 0.007; 68.4% vs 67.8% molar), but lower concentration of propionate (p<0.001; 15.4% vs 16.0% molar) than CON silages (Table 6). Rumen pH, ammonia-N, total VFA, iso-butyrate, iso-valerate, valerate, and acetate to propionate ratio were not affected by inoculant application. With increasing DM content of WCR forage, total VFA (p = 0.001), iso-butyrate (p = 0.036), and valerate concentrations (p = 0.008) of WCR silages decreased linearly.

## DISCUSSION

In general, the chemical compositions of fresh WCR forage in the present study were in a normal range according to several previous studies [16,17]. In addition, decreasing EE content of WCR in the present study was associated with increasing level of DM content. A wilting process under the sun to increase DM content evidently could decrease the EE content in the forage due to the oxidation process [18,19], which was in agreement with the result of EE in the present study. After ensiling, pH and organic acid from all silages were similar to the previous studies [20,21] and represented high quality of fermentation, which could inhibit nutrient loss during fermentation in WCR silage (Table 3). This might be a reason for the lack of an effect of inoculant application on CP, NDF, ADF, HEMI of WCR silage. Low pH condition inhibited the growth of undesirable microbes and stopped the natural enzymes from degrading protein and carbohydrate during ensiling [9]. In addition, the result of EE content after ensiling followed the result of EE content before ensiling because the condition in all silages were acid enough to inhibit the lipid oxidation during ensiling [9,19]. Thus, only the effect of DM content affected the EE content after ensiling without any effect from inoculant. On the other side, a lower DM content of WCR after ensiling than before ensiling in all treatments indicated that soluble carbohydrate was used by silage microbes to grow and produce organic acid [9]. The lose of DM content during ensiling could change the percentage of the other chemical composition in silages. Therefore, this was a reason of increasing content of other chemical compositions such as CP, EE, NDF, ADF, and HEMI in WCR after ensiling.

The WCR silage had pH range at 4.40 to 5.00 in previous studies [20,21], which was like the results in the present study. The lower pH in CON silages than in INO silages were caused by high lactate production, which lactate had highest degree of acidification compared with acetate, propionate or butyrate [9,22]. Inoculants in the present study, *L. brevis* 5M2 and *L. buchneri* 6M1, are classified as hetero fermentative LAB, where these LAB could convert the lactate into acetate or propionate [9]. This was a reason for the lower lactate concentration and higher concentrations of acetate and propionate in INO silages than in CON silages. In general, CON silage presented higher ammonia-N and butyrate concentrations than INO silage in all of DM contents. The presence of ammonia-N and butyrate could be indicators of the nutrient degradation by undesirable microbes [9,22]. Moreover, the presence of those compounds would decrease feed palatability for ruminant [9,23]. The higher production of antimicrobial compounds including acetate and propionate in INO silages inhibited the undesirable microbes such as mold (Table 4), and then could potentially increase the aerobic stability of silage [22,24]. The results of present study were supported by several previous studies that reported decreases of ammonia-N and butyrate concentrations in silage following inoculant application [3,4]. On the other side, silage pH increased with increasing DM content of WCR forages that was appropriate with decreasing lactate and acetate concentrations of WCR silages. Decreasing moisture content decreased organic acid such as lactate and acetate production in the silage because LAB have low activity in low moisture silage [9,25]. This supported the result of LAB count in the present study (Table 4) where LAB numerically decreased with increasing DM content of WCR forages. In addition, ammonia-N concentration also decreased with increasing DM content of WCR forages that was in agreement with decreases of yeast and mold counts in WCR silage (Table 4).

In microbial count, higher LAB count by inoculant appli cation agreed with previous studies [3,4]. The present study reported that mold count also decreased by inoculant application, which confirmed an antifungal activity of *L. brevis* 5M2 and *L. buchneri* 6M1. Similar with the present study, application of antifungal-producing inoculant could inhibit the growth of mold in silage [26]. The increasing DM content of WCR forages lead to decreases of yeast and mold counts in the present study. Previous studies have shown limitations of growth on those microbes in silage with low moisture content [9,25].

In general, rumen degradation kinetics reflects the result of the rumen digestibility [27]. In the present study, the immediately degradable fraction decreased by inoculant application that supported the increases of LAB count (Table 4) and total organic acid (lactate and acetate; Table 3) in INO silages. A high population of LAB in INO silages increased the use of the immediately degradable fraction, such as soluble carbohydrate during ensiling to support their maintenance and produce more organic acid [9]. Thus, remain of soluble carbohydrate was lower in INO silages than in CON silages. However, the application of *L. brevis* 5M2 and *L. buchneri* 6M1 on WCR silage had proven to improve nutrient digestibility by increase degradation of the potentially degradable fraction and the total degradable fraction in the rumen. Both these LABs produce carboxylesterase that could break down the lignin complex, and then increased the accessibility of other fibrinolytic enzymes to degrade structural carbohydrate in the silage [2]. Similar to the present study, several previous studies also reported the improvement of rumen digestibility by application of inoculant producing esterase [4,5]. The reason for the lower the fractional degradation rate in INO silages was unclear. The LAB from silage could pass and survive in the rumen ecosystem, but their interaction with rumen microbes digesting the silage was unknown [27,28]. The interaction between silage inoculant and rumen microbes resulted in the lower rate of fractional degradation such as the result of INO silages in the present study. Nevertheless, the low fractional degradation rate didn’t always influence the total degradable fraction. On the other side, higher total degradable fraction in INO silages also supported with higher total VFA and acetate concentration than in CON silages. Increasing concentration of total VFA with a high acetate proportion indicated a high digestibility of structural carbohydrate in the rumen [27,29], where structural carbohydrate was the main chemical composition of WCR silage. High proportion of acetate in the rumen could decrease the propionate proportion [27,29], which supported the result of INO silages in the present study. The previous study reported that increasing DM content at ensiling had no effect on digestibility and fermentation characteristics of alfalfa silage in the rumen [25]. However, Santos and Kung [30] reported that low DM content at ensiling had higher rumen NDF digestibility than high DM content, which was in agreement with the result of the present study. With increasing DM content of WCR forage, the potentially and total degradable fractions decreased that supported the decreases of total VFA, iso-butyrate, and valerate concentrations in the present study. These results indicated that the nutrient digestibility of WCR silage decreased with increasing DM content of WCR forage.

## CONCLUSION

The present study concluded that application of antifungal and carboxylesterase-producing inoculant on WCR silage improved acetate concentration, mold inhibition, and rumen digestibility in all DM contents. Mold was not detected when DM was at haylage levels in either CON or INO silages. With increasing DM content of WCR forage, fermentation indices and rumen digestibility of WCR silage decreased. The present study confirmed that antifungal and esterase activities of the inoculants were effective at the DM levels for making silage or intermediate between silage and haylage.

## Figures and Tables

**Table 1:** Chemical compositions of whole crop rice before ensiling (%, DM)

Items	CON1)			INO1)			SEM
	
	LOW2)	MED2)	HIGH2)	LOW2)	MED2)	HIGH2)	
DM	35.1	43.6	51.5	35.7	43.7	51.6	0.587
CP	8.83	8.94	8.87	8.80	8.89	8.61	0.307
EE	2.66	2.56	2.36	2.66	2.57	2.38	0.143
CA	7.73	7.60	7.91	7.68	7.90	7.38	0.627
NDF	50.0	49.9	49.8	50.3	49.9	50.4	0.616
ADF	28.4	27.8	27.5	27.9	27.9	28.2	0.665
HEMI	21.5	22.1	22.3	22.4	22.0	22.2	0.879
	
Contrast	**DM**	**CP**	**EE**	**CA**	**NDF**	**ADF**	**HEMI**
	
Inoculant	0.300	0.441	0.769	0.737	0.305	0.593	0.572
DM content	<0.001	0.672	<0.001	0.990	0.731	0.715	0.739
Inoculant×DM content	0.648	0.693	0.992	0.491	0.646	0.274	0.603
DM content linear	<0.001	0.660	<0.001	0.970	0.843	0.453	0.457
DM content quadratic	0.863	0.412	0.217	0.825	0.431	0.715	0.811

DM, dry matter; SEM, standard error of mean; CP, crude protein; EE, ether extract; CA, crude ash; NDF, neutral detergent fiber; ADF, acid detergent fiber; HEMI, hemicellulose.

1) CON, silage without inoculant; INO, silage inoculated with mixture of *Lactobacillus brevis* 5M2 and *Lactobacillus buchneri* 6M1 at ratio 1:1.

2) LOW, forage without wilting; MED, forage wilted for intermediate DM content; HIGH, forage wilted for high DM content.

**Table 2:** Effects of dual-purpose inoculant and DM content on chemical compositions of whole crop rice silage ensiled for 108 d (%, DM)

Items	CON1)			INO1)			SEM
	
	LOW2)	MED2)	HIGH2)	LOW2)	MED2)	HIGH2)	
DM	32.3	39.8	49.3	32.5	39.3	49.0	1.751
CP	8.97	8.96	9.09	9.12	8.97	9.03	0.186
EE	3.28	3.29	3.01	3.28	3.22	3.03	0.095
CA	8.97	8.72	8.99	9.13	8.59	9.03	0.552
NDF	55.0	56.1	55.5	54.6	54.8	53.8	1.396
ADF	32.0	31.8	31.4	32.0	31.8	31.0	1.562
HEMI	22.9	24.3	24.3	22.6	23.0	22.8	0.891
	
Contrast	**DM**	**CP**	**EE**	**CA**	**NDF**	**ADF**	**HEMI**
	
Inoculant	0.855	0.656	0.859	0.942	0.622	0.844	0.204
DM content	<0.001	0.745	0.001	0.459	0.608	0.667	0.433
Inoculant×DM content	0.933	0.456	0.708	0.908	0.742	0.963	0.346
DM content linear	<0.001	0.823	<0.001	0.937	0.942	0.342	0.448
DM content quadratic	0.073	0.375	0.042	0.170	0.326	0.829	0.403

DM, dry matter; SEM, standard error of mean; CP, crude protein; EE, ether extract; CA, crude ash; NDF, neutral detergent fiber; ADF, acid detergent fiber; HEMI, hemicellulose.

1) CON, silage without inoculant; INO, silage inoculated with mixture of *Lactobacillus brevis* 5M2 and *Lactobacillus buchneri* 6M1 at ratio 1:1.

2) LOW, forage without wilting at ensiling; MED, forage wilted for intermediate DM content at ensiling; HIGH, forage wilted for high DM content at ensiling.

**Table 3:** Effects of dual-purpose inoculant and DM content on fermentation indices of whole crop rice silage ensiled for 108 d

Items	CON1)			INO1)			SEM
	
	LOW2)	MED2)	HIGH2)	LOW2)	MED2)	HIGH2)	
pH	4.55	4.68	4.73	4.73	4.75	4.84	0.054
Ammonia-N (% DM)	0.14	0.08	0.06	0.12	0.07	0.06	0.014
Lactate (% DM)	5.69	5.38	4.31	3.84	3.70	2.71	0.635
Acetate (% DM)	4.88	2.70	2.01	8.77	6.66	6.14	0.618
Propionate (% DM)	0.00	0.00	0.00	0.50	0.52	0.41	0.054
Butyrate (% DM)	1.24	0.37	0.01	0.51	0.02	0.00	0.182
Lactate:acetate	1.17	1.99	2.14	0.44	0.56	0.44	0.469
	
Contrast	**pH**	**Ammonia-N**	**Lactate**	**Acetate**	**Propionate**	**Butyrate**	**LA:AC**
	
Inoculant	0.025	0.102	<0.001	<0.001	<0.001	0.022	<0.001
DM content	0.005	<0.001	0.012	<0.001	0.017	<0.001	0.079
Inoculant×DM content	0.314	0.566	0.941	0.932	0.160	0.024	0.092
DM content linear	0.021	<0.001	0.068	0.030	0.593	<0.001	0.269
DM content quadratic	0.870	0.118	0.462	0.654	0.689	0.059	0.641

DM, dry matter; SEM, standard error of mean; LA:AC, lactate to acetate ratio.

1) CON, silage without inoculant; INO, silage inoculated with mixture of *Lactobacillus brevis* 5M2 and *Lactobacillus buchneri* 6M1 at ratio 1:1.

2) LOW, forage without wilting at ensiling; MED, forage wilted for intermediate DM content at ensiling; HIGH, forage wilted for high DM content at ensiling.

**Table 4:** Effects of dual-purpose inoculant and DM content on microbial count of whole crop rice silage ensiled for 108 d (log10 cfu/g)

Items	CON1)			INO1)			SEM
	
	LOW2)	MED2)	HIGH2)	LOW2)	MED2)	HIGH2)	
Lactic acid bacteria	7.26	7.29	7.13	8.24	7.86	7.85	0.197
Yeast	8.00	7.97	7.87	8.01	7.97	7.83	0.143
Mold	3.25	3.05	ND	ND	ND	ND	0.043
	
Contrast		**Lactic acid bacteria**		**Yeast**		**Mold**	
	
Inoculant		<0.001		0.708		<0.001	
DM content		0.053		0.157		<0.001	
Inoculant×DM content		0.133		0.914		<0.001	
DM content linear		0.271		0.042		0.046	
DM content quadratic		0.844		0.731		0.322	

DM, dry matter; SEM, standard error of mean; ND, not detected.

1) CON, silage without inoculant; INO, silage inoculated with mixture of *Lactobacillus brevis* 5M2 and *Lactobacillus buchneri* 6M1 at ratio 1:1.

2) LOW, forage without wilting at ensiling; MED, forage wilted for intermediate DM content at ensiling; HIGH, forage wilted for high DM content at ensiling.

**Table 5:** Effects of dual-purpose inoculant and DM content on rumen degradation kinetics of whole crop rice silage ensiled for 108 d

Items	CON1)			INO1)			SEM
	
	LOW2)	MED2)	HIGH2)	LOW2)	MED2)	HIGH2)	
A (mL/g DM)	0.72	0.82	0.83	0.65	0.64	0.71	0.077
B (mL/g DM)	3.75	3.60	3.48	3.97	3.93	3.71	0.087
A+B (mL/g DM)	4.47	4.42	4.31	4.62	4.57	4.42	0.025
C (%/h)	0.08	0.09	0.08	0.08	0.07	0.07	0.007
L (h)	1.41	1.42	2.78	1.16	1.08	2.85	0.258
	
Contrast	**A**	**B**		**A+B**	**C**	**L**	
	
Inoculant	0.032	0.001		<0.001	0.011	0.293	
DM content	0.359	0.004		<0.001	0.147	<0.001	
Inoculant×DM content	0.600	0.606		0.439	0.067	0.530	
DM content linear	0.255	0.024		0.011	0.326	<0.001	
DM content quadratic	0.946	0.595		0.371	0.351	0.016	

DM, dry matter; SEM, standard error of mean; A, the immediately degradable fraction; B, the potentially degradable fraction; A+B, the total degradable fraction; C, the fraction degradation rate; L, the lag phase.

1) CON, silage without inoculant; INO, silage inoculated with mixture of *Lactobacillus brevis* 5M2 and *Lactobacillus buchneri* 6M1 at ratio 1:1.

2) LOW, forage without wilting at ensiling; MED, forage wilted for intermediate DM content at ensiling; HIGH, forage wilted for high DM content at ensiling.

**Table 6:** Effects of dual-purpose inoculant and DM content on rumen fermentation characteristics of whole crop rice silage ensiled for 108 d

Items	CON1)				INO1)					SEM
	
	LOW2)	MED2)		HIGH2)	LOW2)		MED2)		HIGH2)	
pH	6.43	6.40		6.44	6.41		6.40		6.43	0.027
Ammonia-N (mg/dL)	31.2	27.5		30.1	30.3		29.1		29.1	2.112
Total VFA (m*M*/L)	231.7	231.0		225.4	235.6		233.0		226.5	2.088
Acetate (% molar)	67.4	67.9		68.0	68.2		68.1		69.0	0.412
Propionate (% molar)	15.9	16.2		16.0	15.5		15.7		14.9	0.205
Iso-butyrate (% molar)	1.40	1.34		1.33	1.40		1.41		1.28	0.069
Butyrate (% molar)	12.0	11.5		11.7	11.7		11.6		11.9	0.191
Iso-valerate (% molar)	2.40	2.23		2.21	2.24		2.28		2.25	0.077
Valerate (% molar)	0.98	0.82		0.73	0.88		0.93		0.74	0.100
Acetate:propionate	4.26	4.20		4.26	4.04		4.35		4.65	0.247
	
Contrast	**pH**	**NH_3_-N**	**TVFA**	**AC**	**PR**	**IBU**	**BU**	**IVA**	**VA**	**AC:PR**
	
Inoculant	0.318	0.958	0.097	0.007	<0.001	0.857	0.621	0.719	0.761	0.299
DM content	0.170	0.228	0.005	0.056	0.044	0.098	0.280	0.268	0.025	0.134
Inoculant×DM content	0.731	0.613	0.639	0.337	0.023	0.354	0.194	0.096	0.293	0.184
DM content linear	0.320	0.406	0.001	0.074	0.407	0.036	0.720	0.143	0.008	0.061
DM content quadratic	0.064	0.094	0.138	0.501	0.106	0.501	0.173	0.779	0.349	0.888

DM, dry matter; SEM, standard error of mean; VFA, volatile fatty acid; NH_3_-N, ammonia-N; TVFA, total volatile fatty acid; AC, acetate; PR, propionate; IBU, iso-butyrate; BU, butyrate; IVA, iso-valerate; VA, valerate; AC:PR, acetate to propionate ratio.

1) CON, forage ensiled without inoculant; INO, forage ensiled with mixture of *Lactobacillus brevis* 5M2 and *Lactobacillus buchneri* 6M1 at ratio 1:1.

2) LOW, forage without wilting at ensiling; MED, forage wilted for intermediate DM content at ensiling; HIGH, forage wilted for high DM content at ensiling.
